# Process Optimization of Dual-Liquid Casting and Interfacial Strength–Toughness of the Produced LAS/HCCI Bimetal

**DOI:** 10.3390/ma16052008

**Published:** 2023-02-28

**Authors:** Zhen-Guo Xing, Li-Xin He, Shun-Xing Liang, Lian-Bo Chang, Zhi-Xia Xiao, Wan-Li Xing, Hai-Bin Shen, Jing-Jing Cao, Hong-Ji Liu

**Affiliations:** 1 College of Materials Science and Engineering, Hebei University of Engineering, Handan 056038, China; 2 Handan Huiqiao Compound Material Technology Co., Ltd., Handan 056038, China; 3 Hebei Key Laboratory of Wear-Resistant Metallic Materials with High Strength and Toughness, Hebei University of Engineering, Handan 056038, China; 4 School of Water Conservancy and Hydroelectric Power, Hebei University of Engineering, Handan 056038, China; 5 College of Materials Science and Engineering, Hebei University of Technology, Tianjin 300401, China; 6 College of Mechanical and Equipment Engineering, Hebei University of Engineering, Handan 056038, China

**Keywords:** dual-liquid casting, bimetallic composites, pouring time interval, interfacial protective agent, interfacial strength–toughness

## Abstract

The pouring time interval is the decisive factor of dual-liquid casting for bimetallic productions. Traditionally, the pouring time interval is fully determined by the operator’s experience and on-site observation. Thus, the quality of bimetallic castings is unstable. In this work, the pouring time interval of dual-liquid casting for producing low alloy steel/high chromium cast iron (LAS/HCCI) bimetallic hammerheads is optimized via theoretical simulation and experimental verification. The relevancies of interfacial width and bonding strength to pouring time interval are, respectively, established. The results of bonding stress and interfacial microstructure indicate that 40 s is the optimum pouring time interval. The effects of interfacial protective agent on interfacial strength–toughness are also investigated. The addition of the interfacial protective agent yields an increase of 41.5% in interfacial bonding strength and 15.6% in toughness. The optimum dual-liquid casting process is used to produce LAS/HCCI bimetallic hammerheads. Samples cut from these hammerheads show excellent strength–toughness (1188 Mpa for bonding strength and 17 J/cm^2^ for toughness). The findings could be a reference for dual-liquid casting technology. They are also helpful for understanding the formation theory of the bimetal interface.

## 1. Introduction

Due to its excellent wear resistance, high-temperature strength, and heat resistance, high chromium cast iron (HCCI) has been regarded as the best wear-resistant material [1,2,3]. It has been widely applied in mechanical and metallurgical equipment for crushing, grinding, material handling, and so on. The main applications contain hammerheads, lining plates, grinding balls, flow passage components of mortar pumps, etc. Although components made by single HCCI have certain wear resistance and toughness under ordinary working conditions, it is difficult for them to meet the requirements of high strength, elevated wear resistance, and great security under severe conditions. The steel/high chromium cast iron (S/HCCI) bimetal composites were developed and used to prepare wear-resistant components of which the comprehensive performance is obviously improved [4,5,6].

Most S/HCCI bimetallic components are produced using casting methods. There are two kinds of casting technologies: dual-liquid and liquid–solid. Compared to the liquid–solid, the dual-liquid technology can obtain castings with a better interfacial region and performance, and have a low cost as well [7]. Thus, dual-liquid casting is the main technology for the production of bimetallic castings [8,9]. Previous works [9,10,11,12] have proved that the pouring temperature and interval between two pours greatly affect the interfacial microstructure, bonding strength, and the whole strength–toughness of bimetallic castings. However, most previous reports mainly focus on casting technology. Some prior works have investigated the interfacial microstructure evolution and mechanical properties [13,14,15]. However, some theoretical issues, such as key factors of interfacial microstructure evolution, relevance of microstructure and casting process, correlation between interfacial microstructure, bonding strength, and the whole strength–toughness, are still unclear and need to be investigated in order to guide actual production and improve the performance of castings.

This work aims to study the effects of pouring time interval and the interfacial protective agent of dual-liquid casting technology on bimetallic strength–toughness by using theoretical simulation and experiment. The interfacial solidification process, microstructure, bonding strength, and the whole mechanical properties of the LAS/HCCI bimetal prepared using different dual-liquid casting processes are systematically investigated. The findings would act as important guidance for the production of bimetallic casting. They are also helpful to understand the theory of interfacial microstructure evolution.

## 2. Materials and Methods

The bimetallic hammerhead casting is composed of low alloy steel and high chromium cast iron. The ductile low alloy steel, GS-42CrMo4, is used to prepare the handle part, and the wear-resistant high chromium cast iron, G-X300CrMo153, is employed to make the head part. The chemical compositions of these two parts are listed in Table 1. 

**Figure 1:**
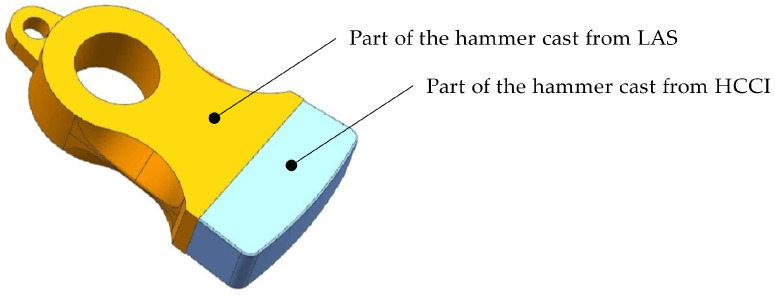
A simulated LAS/HCCI bimetallic hammerhead.

The pouring time interval is the key factor of the interfacial bonding for dual-liquid cast bimetallic hammerheads. Currently, the pouring time interval is usually determined by observing the interfacial solidification degree during actual production. This method has obvious randomness and depends on the operator’s experience. Overlong time intervals would cause a narrow and weak bonding interface. Conversely, short time intervals lead to a wide and even composition mixing layer. This results in unstable quality and performance of the hammerhead castings. In this work, the LAS/HCCI bimetallic hammerhead casting, as Figure 1 shows, is used as the research object to obtain the optimum pouring time interval. through simulating the interfacial temperature variation and solidification degree after the first pouring, the mathematical model to realize a metallurgical fusion layer can be established, and an optimum pouring time interval can be determined.

**Figure 2:**
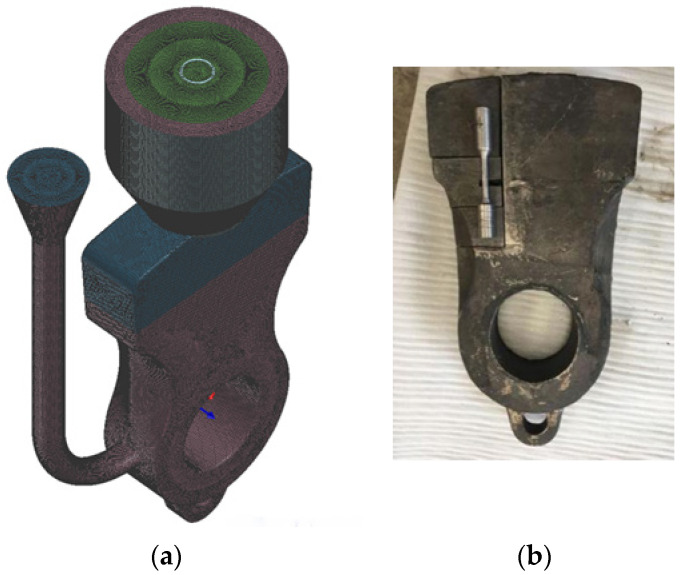
(**a**) Horizontal–vertical casting modeling, and (**b**) a typical bimetallic hammerhead.

LAS/HCCI bimetallic hammerheads are produced at the Handan Huiqiao Composite Material Technology Co., Ltd. using dual-liquid casting technology. The self-setting resin sand process and horizontal molding–vertical pouring technology, as Figure 2a shows, are used during dual-liquid casting. Two medium-frequency fast smelting furnaces are used simultaneously to melt the LAS and HCCI. The raw materials are heated to the designed pouring temperatures of 1576 °C for the LAS and 1400 °C for the HCCI. A self-made interface protective agent was added, according to the actual process, after 10 s of the first LAS pouring. The second HCCI pouring was performed after a certain time interval to the LAS. Pouring time intervals of 35, 40, 45, and 50 s were used to produce the bimetallic hammerhead. After cooling and cleaning, the bimetallic hammerhead castings underwent the following heat treatments: annealing at 1000 °C, tempering at 980 °C, and finally low temperature tempering at 260 °C. All test samples were cut from the heat-treated bimetallic hammerhead castings, as Figure 2b shows.

A scanning electron microscope, SEM, was employed to observe the microstructure of the bimetallic castings. An energy dispersive spectrometer, EDS, was used to measure the distribution of the alloying elements. The line and face scan of EDS were adopted to analyze the variation in the alloying elements around interfacial regions. The accelerating voltage and working distance of SEM and EDS measurements were 10 KV and 6.275 mm, respectively. The detection signal was secondary electrons for the SEM images and backscattered electrons for the EDS analysis. 

**Figure 3:**
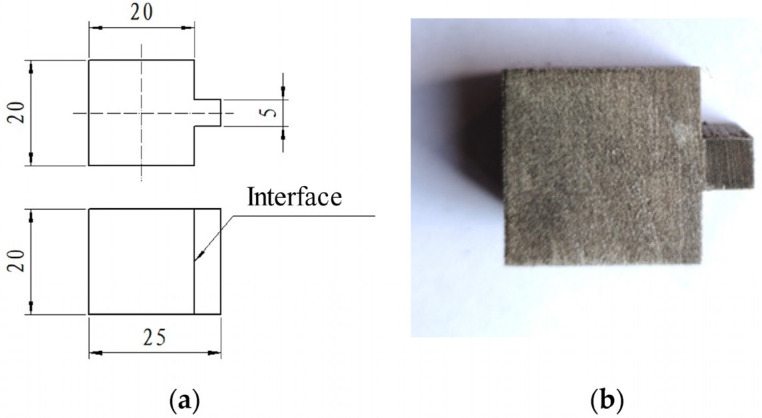
Samples for shear tests: (**a**) schematic drawing and (**b**) physical photo.

The interfacial bonding strength was determined using the shearing test. The schematic drawing and the physical photo of samples for the shear tests are shown in Figure 3. The bulges of shearing samples are the LAS, and the body is the HCCI. The force was loaded continuously on bulges until the fracture, while the body was supported using a special tool. A full-automatic Vickers hardness tester was utilized to measure the hardness. The total force of 1 kg was loaded onto samples and kept for 10 s. To evaluate the hardness variation, tests were performed nearby and across the interfacial region.

**Figure 4:**
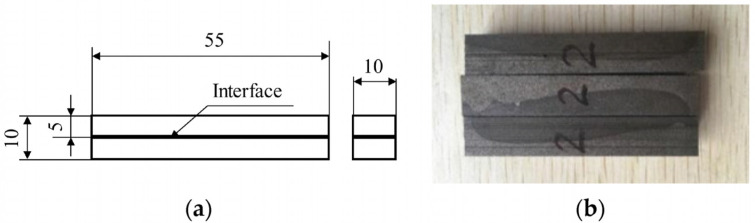
Samples for impact tests: (**a**) schematic drawing and (**b**) physical photo.

**Figure 5:**
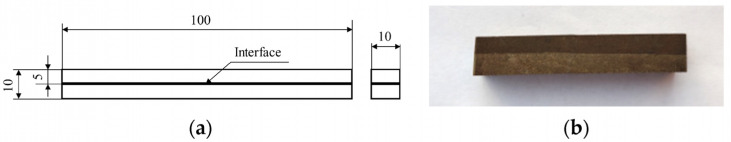
Samples for bending tests: (**a**) schematic drawing and (**b**) physical photo.

**Figure 6:**
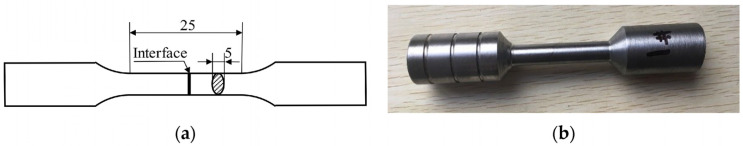
Samples for tensile tests: (**a**) schematic drawing and (**b**) physical photo.

Charpy impact, three-point bending, and tensile tests were employed to determine the overall mechanical properties of the LAS/HCCI bimetal. These test methods were performed according to the standard of ASTM A370-22 [16]. The span of the three-point bending tests was 40 mm. Schematic drawings and physical photos of samples for these tests are shown in Figure 4, Figure 5 and Figure 6, respectively. 

## 3. Results and Discussion

### 3.1. Simulation Results of Interfacial Solidification for Different Pouring Time Intervals

**Figure 7:**
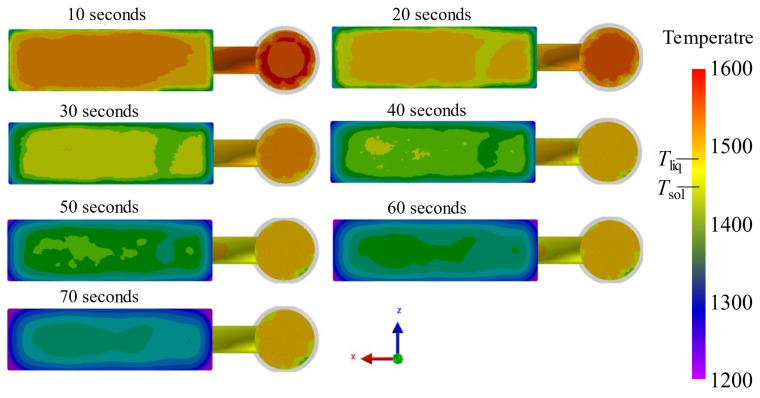
Simulated interfacial temperature field for different pouring time intervals.

**Figure 8:**
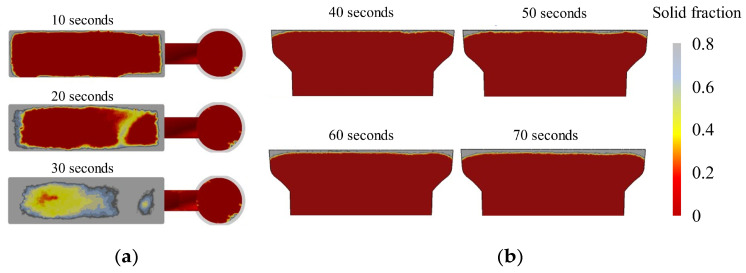
Simulated interfacial solid fraction for different pouring time intervals: (**a**) top and (**b**) side views.

Simulation results regarding the interfacial solidification process of the bimetal are shown, respectively, in Figure 7 and Figure 8. The simulated temperature field results of the interface after the LAS casting (Figure 7) show that the temperature decreases from the center to the edges of the interfacial region. Although the temperature at the edges of the interfacial region after 10 s of pouring is lower than the solidification temperature, the temperature in the central area is far higher than the solid point. As the time interval increases, the temperature in the central area decreases. While the time interval is 40 s, the temperature of the whole interfacial region is lower than the solidification point. This means that the whole interfacial region has gone through solidification already. As the time interval further increases, the temperature of the central area decreases continuously. The temperature of the central area is below 1400 °C while the time interval is 70 s. To further show the interfacial solidification process, the solid fraction in the interfacial region for different time intervals was also calculated, and the results are shown in Figure 8. The solid fraction of the central area in the interfacial region of all samples with a time interval below 40 s is lower than that of the edges. While the interval is higher than 40 s, the whole interfacial region is solidified. This is in accordance with the simulated temperature field results. Furthermore, the thickness of the solidified layer increases with the time interval. When the interval is up to 70 s, the thickness of the solidified layer increases to approximately 5 mm. Previous results [17,18] have indicated that the second casting should be performed at the moment that the interface of the first casting has just reached full solidification. So, the simulation results indicate that the optimum pouring time interval of these bimetallic hammerhead castings is 40 s.

### 3.2. Optimum Pouring Time Interval Verified by Experiment

#### 3.2.1. Pouring Time Interval Dependence on Interfacial Width

**Figure 9:**
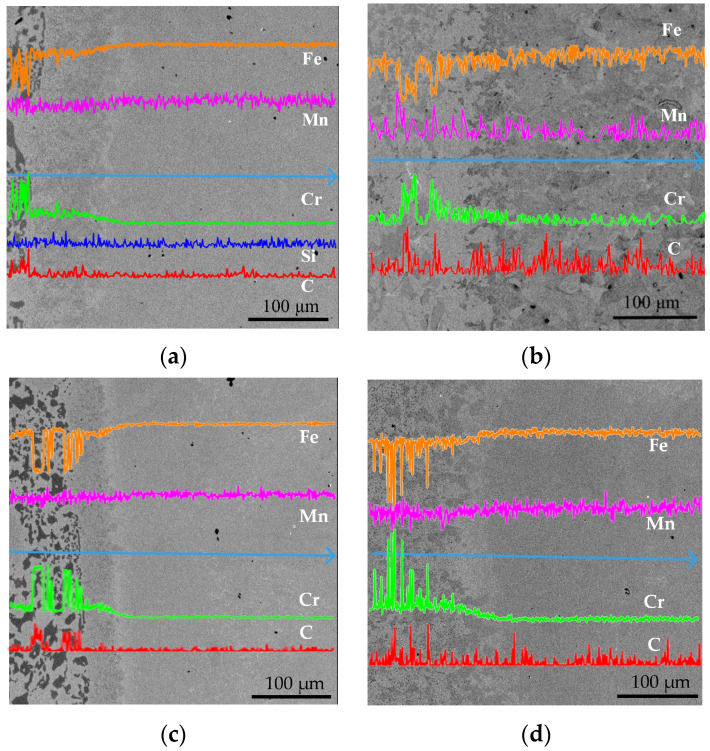
EDS images and element line analysis results of specimens for different pouring time intervals: (**a**) 35 s, (**b**) 40 s, (**c**) 45 s, and (**d**) 50 s.

**Figure 10:**
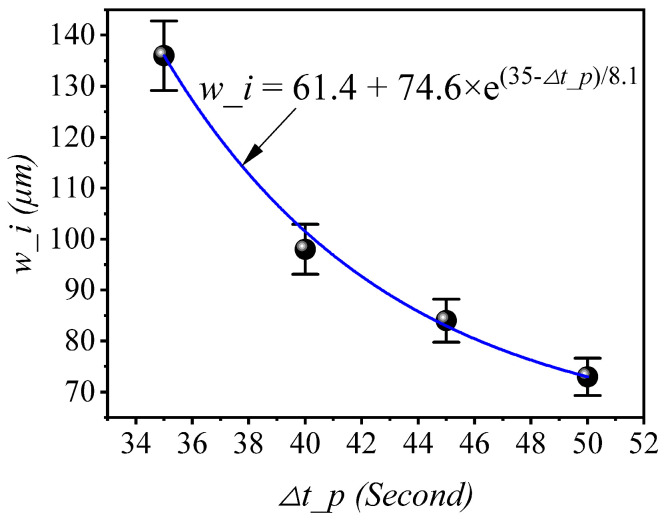
Relevance of interfacial width (*w_i*) to pouring time interval (*∆t_p*).

Figure 9 shows the EDS images and line distributions of alloy elements in specimens for different pouring time intervals. EDS images show different regions for each specimen: the low alloy steel region, the interfacial region, and the high chromium cast iron region. The element diffusion would occur when the concentration difference exists between the two regions. Additionally, diffusion would become more obvious as the temperature and concentration difference increase. The contents of Cr, Mo, and C in HCCI are significantly higher than that in LAS, as Table 1 listed. Thus, these elements diffused from the HCCI side to LAS. However, the absolute content is low at 2.11 wt.% for Mo and 2.73 wt.% for C. In fact, the determination of the C content has low accuracy for ESD. Apart from Cr content, the concentration difference in the solvent element Fe between the two sides is also remarkable and can be easily detected. So, obvious changes in both the contents of Cr and Fe can be detected and shown. Observed drastic changes in elements of Fe, Cr and C in the HCCI side result from inhomogeneity and carbide precipitation. Element distribution results show the gradual changes in the content of elements, especially Fe and Cr, in the interfacial region. Therefore, the interfacial width of dual-liquid cast bimetals can be inferred by measuring the line distribution of alloy elements. Combining the EDS images and corresponding line distributions of alloy elements, the interfacial widths for different pouring time intervals can be obtained and are listed in Table 2. From the width results, it is obvious that the interfacial width decreases with an increasing pouring time interval. The maximum interfacial width is 136 μm for specimen S1, and the minimum interfacial width is 73 μm for S4. So, the interfacial width decreases with an increasing pouring time interval. The variation in the interfacial width obeys the diffusion laws. That is, the diffusion distance increases with temperature and time. Both the temperature of the first casting and the diffusion time decrease with an increasing pouring time interval. So, the diffusion distance, namely *w_i* here, decreases with an increasing pouring time interval. Similar results have also been shown in previous publications [12,17,18]. Based on four-point experimental *w_i* values in Table 2, the fitting relevant equation of interfacial width to pouring time intervals, *w_i* = *f*(*∆t_p*), is obtained through mathematical regression and is shown in Figure 10.

#### 3.2.2. Pouring Time Interval Dependence on Interfacial Bonding Strength

**Figure 11:**
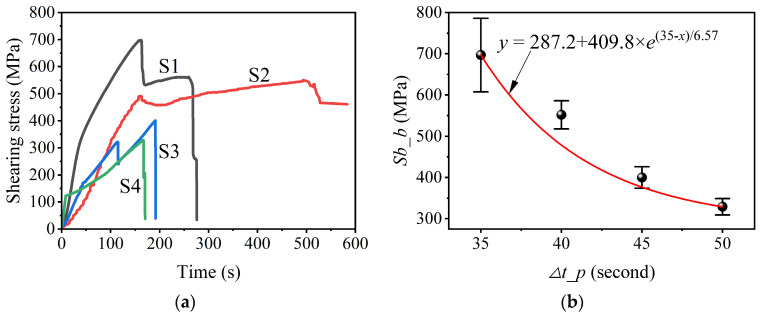
Shearing stress vs. test time curves (**a**) and pouring time interval dependence on interfacial bonding strength (**b**) of different specimens.

Shearing test results of bimetallic specimens produced using different pouring time intervals are shown in Figure 11. From the shearing stress vs. test time curves (Figure 11a), the shearing strength, that can characterize the interfacial bonding strength, of different specimens can be determined. Thus, the interfacial bonding strength of specimens S1 to S4 is, respectively, 697 MPa, 552 Mpa, 400 Mpa, and 329 Mpa. The pouring time interval dependence on the interfacial bonding strength of the LAS/HCCI bimetal is also fitted via mathematical regression based on values of interfacial bonding strength for those four samples and results are shown in Figure 11b. The difference between the interfacial bonding strength of different specimens is mainly because of the variation in interfacial width. Previous publications [19,20,21] indicated that the interfacial bonding strength of bimetals depends on their interfacial width in a certain range. According to the results of Section 3.2.1, the interface width decreases with an increasing pouring time interval. Thus, the interfacial bonding strength decreases with an increasing pouring time interval. Although the interfacial bonding strength increases with the decreasing pouring time interval, a too-short pouring time interval causes the excessive mixing between pairs of bimetals and unstable quality of bimetallic hammerheads, according to actual products and simulation solidification results (see Section 3.2.1). Thus, a pouring time interval of 40 s yields a high interfacial bonding strength, suitable mixing layer, and stable product quality. 

To sum up, the bimetal produced by the process of a pouring time interval of 40 s has a suitable interfacial width, high interfacial bonding strength, and controllable mixing layer. Thus, the optimum pouring time interval is 40 s.

### 3.3. Effect of Interfacial Protective Agent on Interfacial Bonding Strength and Toughness

**Figure 12:**
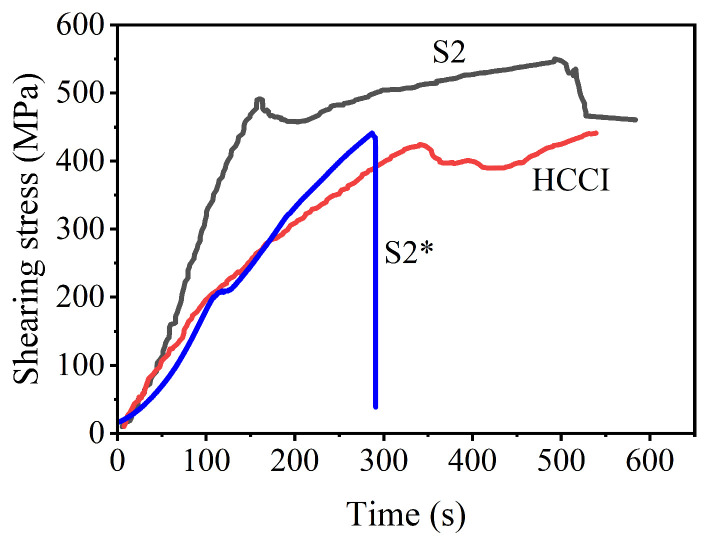
Typical shearing stress vs. test time curves of bimetallic and HCCI samples.

The typical shearing stress–time curves of the bimetallic and HCCI samples are shown in Figure 12. Where the S2 is the sign of the bimetallic specimen produced for the pouring time interval of 40 s with the addition of the interfacial protective agent, S2* is that without adding the interfacial protective agent. Both curves of S2 and HCCI have obvious yielding. However, the sample S2* fractured before yielding. The low yield shearing stress is about 455 MPa for S2, and 390 Mpa for S2* and HCCI. There is a remarkable working hardening and plastic deformation in the curve of S2, but this is inapparent for S2* and HCCI. The maximum shearing stress is approximately 552 Mpa for S2, and 444 Mpa for HCCI. Thus, the bimetallic hammerhead casting with the addition of an interfacial protective agent has higher yield shearing stress, maximum shearing stress, and plastic deformation ability than both the bimetal without the added agent and HCCI. This means that the interfacial bonding strength is 552 Mpa for S2 and 390 Mpa for S2*. An increase of approximately 41.5% in thse interfacial bonding strength is realized after adding the interfacial protective agent. The obvious increase in bonding strength for S2 originates from the cleaning effect and oxidation avoidance of the interfacial protective agent [22]. This interfacial bonding strength of 552 Mpa for S2 is higher than 315 Mpa for a hot diffusion compress bonded HCCI/LCS (low carbon steel) bimetal [5], but is similar to the surface liquid-phase sintered HCCI/LCS bimetal [23]. The key reasons should be the suitable interfacial width and clean interface without oxidation.

The impact toughness values of bimetallic and HCCI samples are listed in Table 3. The average toughness of S2* is 14.7 J/cm^2^. After adding the interfacial protective agent, the toughness of S2 is 17 J/cm^2^, an increase of approximately 15.6% to that of S2*. Thus, the addition of the interfacial protective agent can increase both interfacial bonding strength and toughness. The toughness of S2 is far higher than that of a reported LAS/HCCI bimetal (3.478 to 3.488 J/cm^2^) that is prepared through liquid–solid casting and has a narrow interfacial region [24], and higher than 7.1 J/cm^2^ of a LAS/HCCI bimetal prepared using the hot-rolling process as well [25]. The key reason for such high toughness of specimen S2 would be the suitable interface region and excellent interfacial bonding. This is also proved by the similar toughness (16.9 to 20.2 J/cm^2^) of a carbon steel/HCCI bimetal prepared via the dual-liquid casting method [26]. For comparison, the impact results of HCCI cut from the same casting are also listed in Table 3. The toughness of S2 exceeds 3.5 times that of HCCI. The high toughness of S2 originates from the high toughness LAS and excellent interface. 

To summarize the abovementioned results, the process of a pouring time interval of 40 s and adding the interfacial protective agent is optimum for the production of LAS/HCCI bimetallic hammerheads. 

### 3.4. Microstructure and Properties of the Bimetal Produced by the Optimum Process

**Figure 13:**
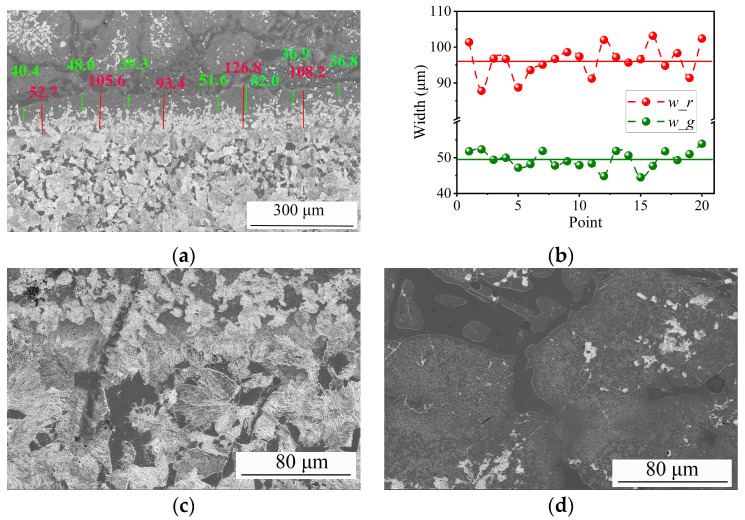
Microstructure near the interfacial region of specimen S2 (**a**) around the interface, (**b**) measured interfacial width data and average values, (**c**) the LAS side, and (**d**) the HCCI side. (**c**,**d**) are enlarged parts of (**a**).

**Figure 14:**
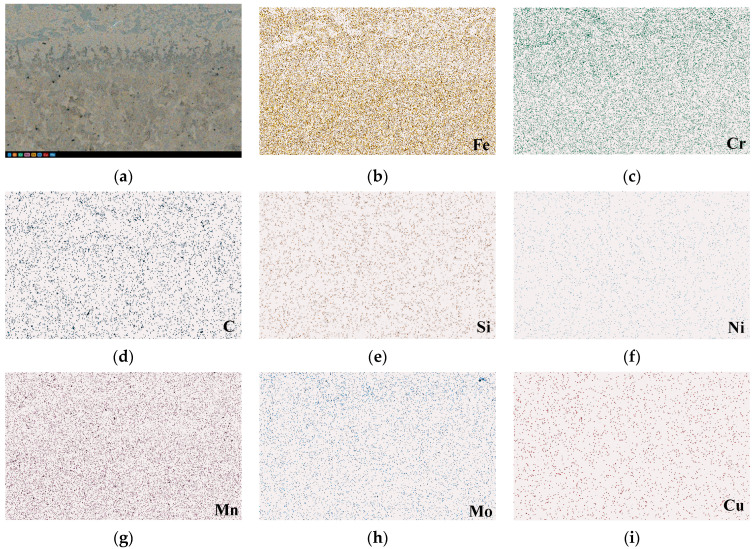
BSD image (**a**) and element distributions (**b**–**i**) in specimen S2.

The microstructural images of the specimen produced by the optimum process (S2) are shown in Figure 13. The microstructure image in Figure 13a also shows an evident interfacial region between the LAS and HCCI regions. Clearer than the EDS image, two different zones can be observed in the interfacial region. These are a fine white precipitation zone and a gray zone that is named a carbide-free zone [27]. Two similar interfacial zones were also reported in previous publications [4,28]. So, we measured the width of the carbide-free zone, the length of green lines, and the width of the whole interfacial region, the length of the red lines. Those green and red lines can be seen in Figure 13a. Hereafter, the markers *w_g* and *w_r* are used, respectively, for the widths of the carbide-free zone and the whole interfacial region. Both *w_g* and *w_r*, namely the length of the green lines and red lines in Figure 13a, are counted and shown in Figure 13b. The *w_g* varies from 44.5 μm to 53.9 μm, and the average *w_g* value is 49.5 μm. The measured *w_r* value varies from 87.8 μm to 103.2 μm, and the average value is 96.3 μm. The interfacial width from the microstructure image is in keeping with that from the EDS analysis listed in Table 2. The microstructures near the interface, of both the LAS and HCCI sides, are also shown, respectively, in Figure 13c,d. The typical microstructure of the LAS side is composed of ferrite plus pearlite, and the microstructure of the HCCI side comprises eutectic chromium carbide in the pearlite matrix. These microstructure features of the LAS/HCCI bimetal are similar to previous reports [4,14,27]. To further confirm the composition of the microstructure in the LAS/HCCI bimetal, the backscattered electron (BSD) image and element distributions of the eight elements, Fe, Cr, C, Si, Ni, Mn, and Mo, around the interface region are measured and are, respectively, shown in Figure 14a–i. The Fe content on the upper side of the EDS image is lower, but the Cr content is higher than that on the lower side. So, the upper side of the EDS image, which is the same as the upper side of Figure 13a, is the HCCI region. This comparison also shows that the dark area in Figure 13d is Cr and C rich, but Fe poor. So, this result can also prove that the dark area in Figure 13d is chromium carbide. 

**Figure 15:**
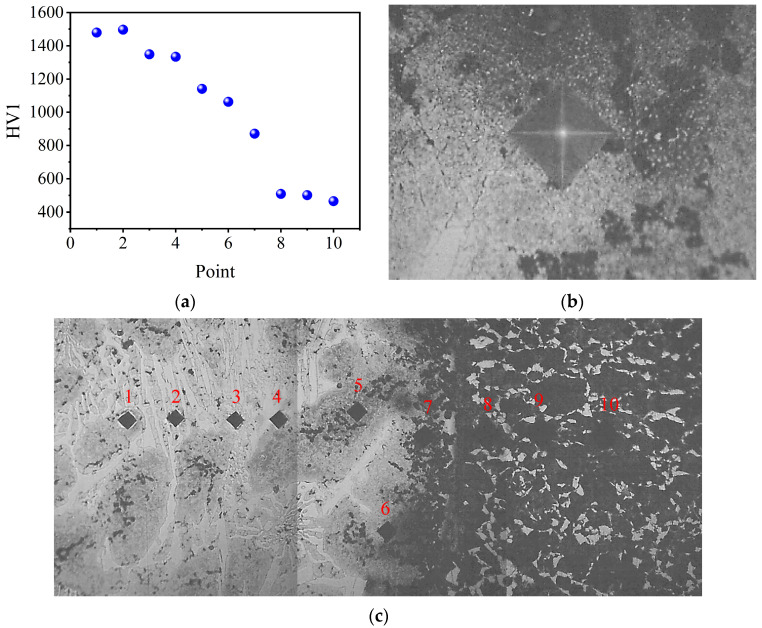
Hardness nearby the interface (**a**), the corresponding indentation of Test Point 6 (**b**), and the locations of all indentations (**c**). The numbers in (**c**) are the testing order which is a one-to-one correspondence with the point of the abscissa in (**a**).

The hardness near the interface of specimen S2 was also tested. The hardness results and corresponding indentations are shown in Figure 15. The HCCI has a high hardness of about 1500 HV1. Additionally, the hardness of the LAS is approximately 500 HV1. Through the interface area, the hardness decreases gradually rather than plunges sharply. This phenomenon is very common in previous publications [14,29,30,31]. The diffusion of elements Cr, C, and Mo from the HCCI side to LAS is the key reason for the decrement in the hardness. Element diffusion from HCCI to LAS increases the content of solute elements gradually in the interfacial region. The increased solute content can realize solid solution strengthening. Additionally, the increased solution content would induce carbide precipitation that can also enhance the hardness. Test Point 6, which is located in the interfacial region, shows a clear indentation. Previous works [32,33,34] indicated that the indentation of a hardness test can characterize the ductility of metals and alloys. The more and/or longer cracks appear, the worse the ductility of metals is. No cracks can be found around the corners of the indentation of Point 6, as Figure 15b shows. This means that the interfacial region of the cast hammerhead has good ductility.

**Figure 16:**
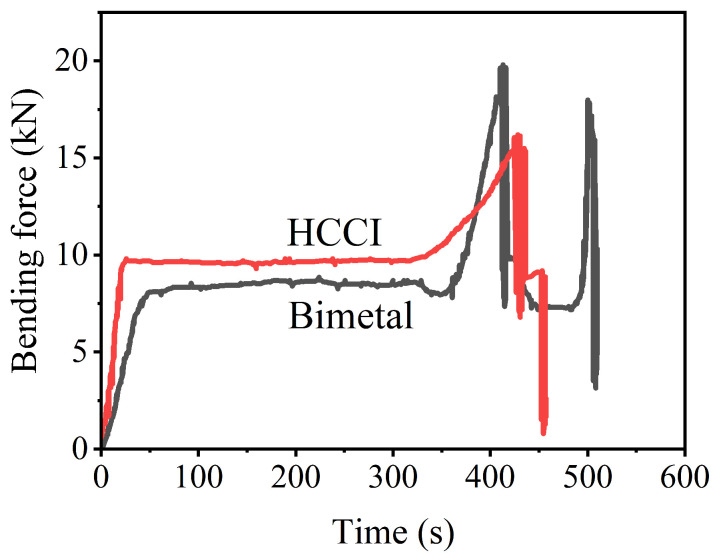
Bending force vs. time curves of the bimetal and HCCI.

The bending force–time curves of the bimetal and HCCI are shown in Figure 16. The maximum bending force is 19.78 kN for the bimetal and 16.11 kN for HCCI. That is, the bending strength is 1188 MPa for the bimetal and 972 Mpa for HCCI. The high bending strength may be because of the energy consumption and working hardening of the LAS. The bending strength of bimetallic samples in this work is far higher than that of high-Cr white cast iron and AISI4140 steel bimetallic beams (650 to 790 Mpa) [35]. The fracture behavior between the bimetal and HCCI samples is also different from each other. The HCCI breaks immediately after the maximum force. However, a second increase in bending force is needed before fracturing the bimetal. This also proves that the fracture of the bimetal needs more external energy than HCCI.

**Figure 17:**
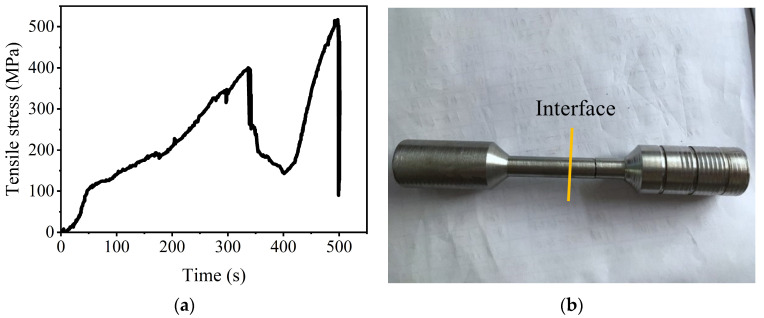
Tensile stress vs. time curve (**a**) and a fractured sample photo (**b**) of the bimetal.

The tensile stress vs. the time curve of the bimetal from a hammerhead casting is shown in Figure 17a. The maximum tensile stress of this curve is about 515 Mpa. This value is higher than 397 Mpa of the high vanadium alloy steel/low carbon steel bimetal [28]. The elevated strength should result from the different raw materials and interfacial bonding strength. The photo of a typical fractured sample is also shown in Figure 17b. The interface of the LAS/HCCI bimetal is nearly in the middle of the sample. However, the fracture occurs on the right side, namely the HCCI side. This phenomenon proves that the bonding strength of the bimetal is excellent and even higher than the HCCI. This fracture phenomenon was also shown in a previous publication [28]. 

In brief, the bimetallic hammerhead produced by the optimum dual-liquid casting process is as follows: a pouring time interval of 40 s with the addition of an interfacial protective agent has a suitable interfacial width of approximately 96.3 μm, excellent bonding strength, and good whole strength–toughness. 

## 4. Conclusions

The effects of the pouring time interval and the interfacial protective agent of the dual-liquid casting process on the interfacial properties of the LAS/HCCI bimetal are studied through theoretical simulation and experiment. The microstructure and properties of bimetallic wear-resistant hammerheads produced using the optimized dual-liquid casting process are also systematically investigated. The main conclusions are as follows.

(1) The interfacial width decreases from 136 μm to 73 μm, and the interfacial bonding strength decreases from 697 MPa to 329 MPa as the pouring time interval increases from 35 s to 50 s. The pouring time interval dependence equations of interfacial width and bonding strength are also established.
(2) Comparing with the specimen produced by the pouring time interval of 40 s without an interfacial protective agent, an increase of 41.5% in interfacial bonding strength and 15.6% in toughness are realized under the positive effect of the interfacial protective agent.
(3) Both theoretical simulation and experiment results indicate that the process with a pouring time interval of 40 s and adding the interfacial protective agent is the optimum dual-liquid casting process to realize metallurgical bonding for the studied LAS/HCCI bimetal.
(4) The interfacial region of the LAS/HCCI bimetal produced by the optimum process is composed of two zones: a fine white precipitation zone and a carbide-free zone. The width of the carbide-free zone and the whole interfacial region is approximately 49.5 and 96.3 μm, respectively. The average toughness and bending strength are, respectively, 17 J/cm^2^ and 1188 MPa. The excellent interfacial bonding strength causes the tensile fracture occurring on the HCCI side, which has the maximum tensile stress of 515 Mpa.

## Figures and Tables

**Table 1:** Normal compositions of the used LAS and HCCI.

Parts	Materials	Normal Chemical Composition, wt.%								
		C	Si	Mn	P	S	Cr	Mo	Ni	Fe
Head	G-X300CrMo153	2.73	0.48	0.65	0.035	0.026	14.66	2.11	0.101	Bal.
Handle	GS-42CrMo4	0.37	0.26	0.85	0.019	0.015	0.81	0.24	0	Bal.

**Table 2:** The interfacial width of specimens for different intervals inferred from EDS.

Specimens	Intervals, s	Width, μm
S1	35	136 ± 12
S2	40	98 ± 9
S3	45	84 ± 9
S4	50	73 ± 8

**Table 3:** Toughness of the studied bimetal and HCCI.

Sample	Toughness (J/cm^2^)			
	1	2	3	Average
S2*	14	13	17	14.7
S2	17	15	19	17
HCCI	5	4.5	5	4.8

## Data Availability

The data presented in this study are available on request from the corresponding author. The data are not publicly available due to privacy.
